# Vocal cord involvement and hoarseness in a patient with Behcets disease: A rare case report

**DOI:** 10.1097/MD.0000000000020221

**Published:** 2020-05-22

**Authors:** Peng Zhou, Qi Wu, Jinnan Li

**Affiliations:** a Otorhinolaryngology-Head and Neck Department; b Pathology Department, West China Hospital, Sichuan University, China.

**Keywords:** Behcets disease, hoarseness, vocal cords

## Abstract

**Rationale:**

Behcets disease (BD) is a type of chronic systemic vasculitis that typically manifests as a mucocutaneous disease with orogenital ulcers, skin damage, and uveitis. The clinical diagnosis is often difficult because of the diversity of organs that may be involved and lack of specific pathological diagnosis.

**Patient concerns:**

A 26-year-old woman presented as a nearly 2-week history of hoarseness with throat pain.

**Diagnoses:**

In the present case, Fiber laryngoscopy showed multiple ulcers involving the epiglottic tubercle, bilateral false vocal cord, middle area of the left vocal cord, and full length of the right vocal cord. Multidisciplinary physicians combined the patients clinical manifestations and pathological findings to make the Behcets disease diagnosis.

**Interventions:**

As the diagnosis confirmed, immediately began appropriate medical therapy (prednisolone at 30 mg once per day and thalidomide at 50 mg once per night in a month).

**Outcomes:**

The ulcer on the right vocal cord disappeared but left a scar. Therefore, the patient experienced only partial recovery from the hoarseness.

**Lessons:**

Behcets disease can cause damage to multiple organs. Although the combination of vocal cord ulcers and hoarseness is rare in patients with BD and has not been previously reported to date, such patients should be treated with caution in clinical practice.

## 1 Introduction

Behcets disease (BD) was named after the Turkish dermatologist Behcet, who published a case report in 1937. Although cases of BD seem to cluster along the ancient Silk Road with the highest prevalence in Turkey, BD also occurs worldwide.^[1]^

Behcets disease is a type of chronic systemic vasculitis that typically manifests as mucocutaneous disease with orogenital ulcers, skin damage, and uveitis. However, involvement of the musculoskeletal system, central nervous system, gastrointestinal tract, vascular beds, kidneys, and cardiopulmonary system can lead to significant morbidity and mortality.^[2]^ The clinical diagnosis is often difficult because of the diversity of organs that may be involved and lack of specific pathological diagnosis. Therefore, it is quite important to identify clinical manifestations and signs as early and comprehensively as possible.

We herein describe an extremely rare case of BD that manifested as hoarseness and vocal cord ulcers as the first signs. Similar cases are limited in the published literature and are worth receiving attention by clinicians.

### 1.1 Consent

Informed consent was signed by the patient for publication of this report and its related images.

### 1.2 Case Presentation

**Figure 1:**
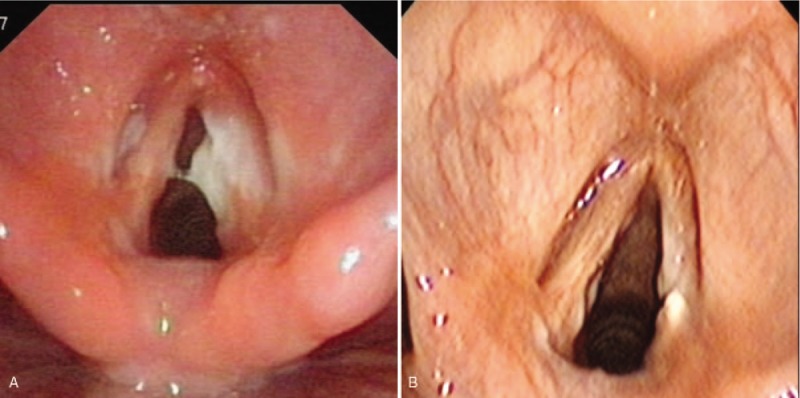
A. The ulcer on the right vocal cord. B. After therapy, the ulcer on the right vocal cord disappeared and only left a scar.

**Figure 2:**
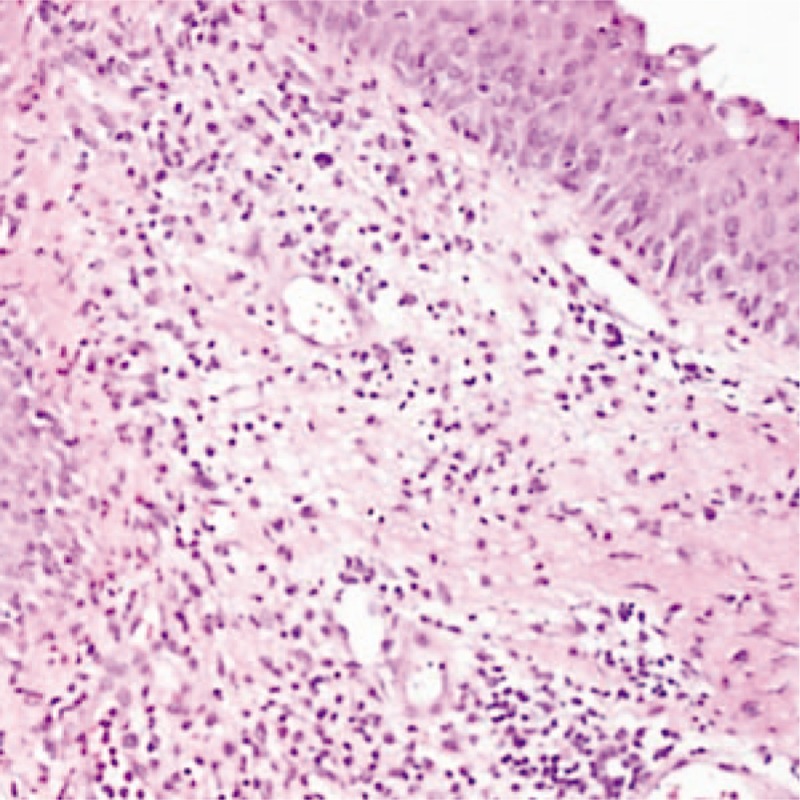
Hematoxylin–eosin staining showed moderate to severe chronic inflammation [activity (++)] with necrosis.

A 26-year-old woman presented to our ear nose throat (ENT) clinic because of a nearly 2-week history of hoarseness with throat pain. The hoarseness of voice was measured by the Grade, Roughness, Breathiness, Asthenia, and Strain (GRBAS) scale (G3R1B1A1S0). Indirect laryngoscopy revealed that the right vocal cord was abnormal. It seemed to have a white film covering, but because of the shield of the epiglottis, it was impossible to see clearly. Fiber laryngoscopy subsequently showed multiple ulcers involving the epiglottic tubercle, bilateral false vocal cord, middle area of the left vocal cord, and full length of the right vocal cord. A white pseudomembrane was attached to the surface of the ulcer, especially on the right vocal cord (Fig. 1A). A biopsy specimen was taken from the right vocal cord ulcer immediately under the fiber laryngoscope to aid in the diagnosis. The primary clinical diagnosis was laryngeal ulceration, special infection (possible laryngeal tuberculosis or laryngeal syphilis). Additional ulcers were found in the oral cavity and on the perineum 1 week later. The pathological examination revealed moderate to severe chronic inflammation [activity (++)] with necrosis, hexammine silver negativity, and detection of *Mycobacterium tuberculosis* and its deoxyribonucleic acid (DNA) fragments by acid staining and polymerase chain reaction for tuberculosis (Fig. 2). The diagnosis of Behcets disease was confirmed based on appropriate clinical symptoms and signs and exclusion of differential diagnoses. Cooperation among multidisciplinary specialists including ENT, rheumatology, ophthalmology, and stomatology experts was mandatory to reach the diagnosis. After 1 month of therapy with prednisolone at 30mg once per day and thalidomide at 50mg once per night, the symptoms and signs were controlled. The ulcer on the right vocal cord disappeared and only left a scar (Fig. 1B). During 3 months follow-up, there was no relapse. The voice further improved in perceptual evaluation, GRBAS scale (G1R1B0A0S0).

## 2 Discussion

Behcets disease occurs worldwide, but clusters are found mainly along the ancient Silk Road extending from eastern Asia to the Mediterranean basin, with the highest prevalence in Turkey.^[3]^ While people aged 20 to 40 years are most commonly affected, BD is also seen in children and older patients. After 55 years of age, however, the diagnosis must be made very cautiously. The incidence is higher in males within the high-prevalence areas of Turkey and the Middle East, but the sex distribution is variable in other counties.^[1,3]^

The pathogenesis of BD is still unclear. The disease may be associated with heredity, immunity, infection, or the living environment. Environmental factors, cytokines, granulocytes, and heat shock protein antigens trigger immune dysfunction and neutrophil hyperfunction in people with genetic susceptibility, leading to vascular endothelial cell injury and dysfunction with related histopathological damage.^[4]^ The diagnosis of BD is based on the clinical picture, which is defined by characteristic features after exclusion of relevant differential diagnoses by appropriate assessments and investigations. Diagnostic clinical manifestations include recurrent oral ulcers; vulvar ulcers; ocular, blood vessel, and nervous system damage; and characteristic skin lesions such as nodular erythema, pseudofolliculitis, papulopustular herpes, and an acne-like rash. The above typical clinical manifestations are highly suggestive of BD. The lack of specific serological examination findings makes the diagnosis of BD difficult. An acupuncture reaction is the only sign with strong specificity and is associated with disease activity.^[2,4]^ Thus, the International Behcets Disease Research Group conducted an in-depth study of 2556 patients with BD from 27 countries in 2013 and proposed a new classification standard and scoring system for diagnosis. New standard scores were established for ophthalmia, oral ulcers, genital ulcers, skin lesions, central nervous system involvement, and vascular involvement. A patient with a total score of ≥4 can be diagnosed with BD. The acupuncture test is not a necessity, but 1 point can be added if the test result is positive.^[5]^ However, the clinical manifestations of hoarseness and vocal cord ulcers described in the present case are not included in the scoring system (Table 1).

**Table 1:**
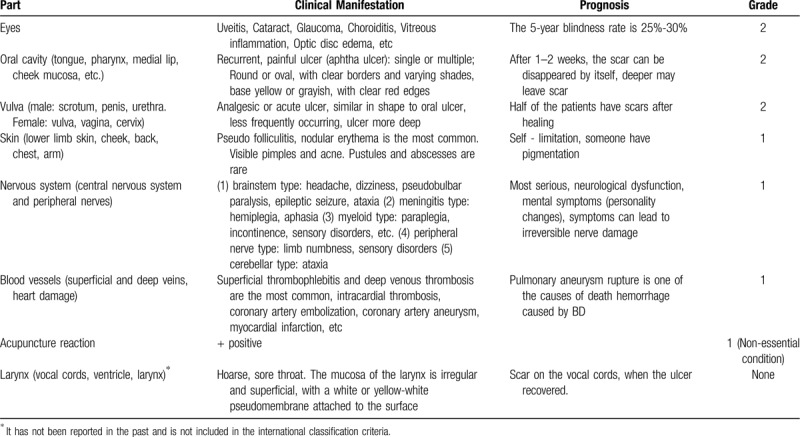
Clinical manifestation, prognosis and international scoring criteria of Behcets disease.

Because so little is known about BD, a huge amount of research on BD has been performed. Because of the complex etiology and multisystem involvement of BD, management is driven by recommendations of expert bodies such as the European League Against Rheumatism, with consensus statements. The purposes of these recommendations and consensus statements are to facilitate functional recovery from visceral involvement and prevent relapse. In general, topical or systemic immunosuppressors and steroids are the basis of anti-inflammatory therapy in patients with BD. Some new biologics have been reported to improve the clinical symptoms and control the disease activity in patients with partially refractory multisystem involvement of BD. Such biologics include apremilast, anakinra, canakinumab, tocilizumab, ustekinumab, infliximab; however, more evidence is needed regarding their efficacy.^[2,6]^

Because laryngeal involvement is a relatively rare manifestation of BD, few case reports have exemplified the potential risks of laryngeal stenosis and the difficulties in treatment, which sometimes includes surgery.^[7,8]^ Hoarseness caused by vocal cord ulceration in patients with BD has not been reported in the literature. In the present case, the diagnosis was confirmed in a timely manner because of early multidisciplinary cooperation, and the clinical manifestations were alleviated after 1 month of conventional treatment. Fortunately, the vocal cord ulcer markedly improved and disappeared, being replaced by a scar. The hoarseness improved but did not completely resolve because the scar damaged the mucosal waves of the vocal cords.

In summary, Behcets disease involving the throat is rare but does exist. Previous reports have focused on complications such as cleft palate and laryngeal lesions with resultant dyspnea. This case report improves our understanding of the clinical features of the laryngeal area, especially the vocal cords, in patients with BD. It is expected to help achieve early diagnosis and treatment of this disease, which can potentially affect voice function and even cause life-threatening disorders.

## 3 Learning points

Behcets disease can cause damage to multiple organs. Although vocal cord ulcers and hoarseness are rare and have not been previously reported, they should be treated with caution in clinical practice.

The diagnosis of Behcets disease is not only dependent upon the pathological and immunological examination findings, but multidisciplinary diagnosis and treatment can help patients to receive early and timely treatment.

## Acknowledgments

The authors thank Dr. Honghu Tang, who assisted with the rheumatologic examination.

## Author contributions

Jinnan Li provided consultation regarding the pathologic findings.

**Visualization:** Peng Zhou, Jinnan Li.

**Writing – original draft:** Peng Zhou, Qi Wu.

**Writing – review & editing:** Peng Zhou.
